# Supplementary material for: Value of the Lymphocyte Transformation Test for the Diagnosis of Drug-Induced Hypersensitivity Reactions in Hospitalized Patients with Severe COVID-19
Source: Int J Mol Sci. 2023 Jul 17;24(14):11543. doi: 10.3390/ijms241411543 (PMC10380365; doi:10.3390/ijms241411543)
Supplement: Supplementary file 1 [file ijms-24-11543-s001.zip › ijms-2502122-supplementary.pdf]

Supplementary Table S1. Cytokine secretion of PBMC. Grey background corresponds to the condition where LTT was positive.

Patient	Treatment	IFNg	IL1B	IL6	TNFa	IL10	IL13	IL4	IL5
**P1**	Control	2.70	0.51	1.45	12.50	2.99	0.71	1.05	0.09
**P1**	HCQ 10	39.65	5.86	18.80	45.14	20.05	113.57	22.14	29.16
**P1**	LOP 5	0.59	0.32	0.87	9.12	1.61	0.02	0.04	0.00
**P1**	RIT 5	0.69	0.50	0.99	14.02	1.75	0.04	0.08	0.00
**P2**	Control	2.69	0.46	1.96	9.17	7.63	0.81	0.22	0.01
**P2**	AZT 0.1	2.87	2.01	13.20	14.32	14.52	0.76	1.02	0.09
**P2**	AZT 1	1.18	0.03	1.36	13.10	9.40	1.02	0.33	0.02
**P2**	AZT 10	1.43	0.06	1.23	11.82	11.70	0.00	0.25	0.01
**P2**	RIT 5	0.84	0.00	0.64	2.88	4.27	0.00	0.00	0.00
**P3**	Control	0.48	0.82	1.43	5.70	2.22	2.14	0.45	0.00
**P3**	CLA 10	3789.92	4.14	36.27	22.06	38.03	2.12	5.50	16.91
**P3**	CLA 100	2085.31	5.87	15.28	12.69	60.84	4.71	6.19	45.23
**P3**	AZT 10	3.90	0.77	0.86	6.80	15.19	1.06	0.31	0.53
**P3**	LOP 5	3.52	0.73	0.74	7.43	13.18	1.60	0.26	0.45
**P4**	Control	0.93	0.82	2.16	0.01	5.10	0.01	0.52	0.02
**P4**	HCQ 10	0.58	0.62	1.56	5.12	3.82	0.51	0.08	0.00
**P4**	LOP 0.2	46.37	1.83	14.14	13.28	10.69	19.96	1.23	21.29
**P4**	RIT 0.2	19.06	1.52	15.29	22.34	9.56	23.42	1.39	23.85
**P5**	Control	1.25	0.06	1.12	27.68	1.89	0.46	0.52	0.01
**P5**	AZT 10	266.52	2.99	10.31	11.61	4.18	2.75	2.31	1.77
**P5**	HCQ 1	79.69	0.58	2.83	10.77	1.65	2.25	1.39	0.83
**P5**	RIT 5	1.18	0.17	1.42	28.85	1.63	0.02	0.08	0.00
**P6**	Control	2.70	0.35	0.85	0.00	5.80	2.09	0.89	0.00
**P6**	AZT 0.1	2.22	0.15	0.83	14.59	4.66	2.01	1.06	0.00
**P6**	LOP 5	4.12	0.33	0.79	3.79	3.91	0.22	0.13	0.00
**P6**	RIT 0.2	306.96	0.60	24.39	15.99	32.75	4.44	7.30	548.30
**P7**	Control	3.79	1.09	8.36	76.47	9.32	2.32	1.86	0.21
**P7**	HCQ 10	4.47	16.46	14.05	53.79	4.33	0.10	0.41	0.11
**P7**	LOP 5	272.41	2.69	14.64	105.50	10.34	3.69	2.01	8.04
**P7**	RIT 5	325.94	53.22	297.45	116.41	17.67	22.62	2.96	8.02
**P8**	Control	3.28	2.60	16.07	55.23	5.75	2.27	1.23	1.08
**P8**	HCQ 10	224.03	3.24	8.77	13.55	2.89	0.57	0.22	0.78
**P8**	LOP 1	3.51	23.14	163.08	113.46	11.22	3.11	3.03	109.75
**P8**	RIT 5	1007.90	37.16	113.33	108.12	11.72	69.17	4.79	79.15
**P9**	Control	7.43	59.89	162.59	5.26	2.56	4.04	3.73	1.32
**P9**	AZT 10	12.29	112.00	225.52	3.10	1.04	1.97	0.87	1.36
**P9**	HCQ 10	58.70	390.50	317.98	15.95	3.60	7.75	5.31	5.27
**P9**	RIT 5	214.51	422.64	322.48	27.14	3.87	18.71	5.94	7.39
**P10**	Control	21.32	32.28	330.47	35.38	15.77	6.97	6.69	0.98
**P10**	CLA 10	51.42	57.51	996.73	92.84	14.93	6.27	2.09	1.24
**P10**	AZT 10	127.52	18.24	268.48	16.06	10.68	6.80	6.44	1.49
**P10**	RIT 1	192.51	110.40	357.35	96.36	17.25	22.40	6.44	2.37
**P11**	Control	24.39	0.73	2.43	10.04	3.14	2.73	1.23	0.27
**P11**	CLA 10	225.60	2.24	5.61	9.81	2.04	1.60	0.17	0.49
**P11**	LOP 5	1499.82	6.98	19.95	52.93	5.80	40.31	4.79	11.11
**P11**	RIT 5	1712.93	9.49	32.82	85.55	6.13	94.94	5.43	11.90

**NAP1**	Control	1.49	4.17	46.66	7.70	3.79	1.49	1.39	0.03
**NAP1**	CLA 10	64.58	5.99	110.08	6.35	1.86	0.09	0.17	0.14
**NAP1**	AZT 10	83.43	7.95	159.82	9.96	2.49	0.15	0.31	0.17
**NAP1**	HCQ 10	62.12	6.25	117.39	6.19	1.81	0.08	0.17	0.12
**NAP1**	LOP 5	73.80	6.70	111.79	6.38	2.13	0.09	0.17	0.15
**NAP1**	RIT 5	64.40	5.87	122.30	6.92	1.86	0.14	0.22	0.14
**NAP2**	Control	1.61	0.11	1.03	26.34	2.56	2.61	0.71	0.30
**NAP2**	CLA 10	1400.95	6.10	2.18	15.34	1.63	0.80	0.26	0.32
**NAP2**	AZT 10	1404.23	5.61	2.00	11.62	1.50	0.54	0.26	0.31
**NAP2**	HCQ 10	1607.63	6.66	1.97	14.79	1.99	0.71	0.36	0.40
**NAP2**	LOP 5	1355.74	5.48	1.37	13.93	1.61	0.70	0.26	0.36
**NAP2**	RIT 5	1338.73	4.97	1.68	12.26	1.54	0.62	0.22	0.29
**NAP3**	Control	3.53	1.05	8.54	11.75	2.00	2.22	1.06	0.18
**NAP3**	CLA 10	64.19	1.05	25.04	6.96	1.00	0.10	0.08	0.08
**NAP3**	AZT 10	57.06	1.05	21.28	7.34	1.04	0.12	0.08	0.08
**NAP3**	HCQ 10	58.09	1.01	29.72	6.23	1.00	0.11	0.08	0.11
**NAP3**	LOP 5	48.71	0.75	24.38	4.93	0.95	0.07	0.04	0.00
**NAP3**	RIT 5	54.71	0.72	23.03	5.53	0.99	0.10	0.13	0.04
**NAP4**	Control	1.74	0.21	1.07	17.81	1.65	2.78	0.71	0.62
**NAP4**	CLA 10	966.36	10.17	153.57	31.01	3.11	2.37	0.41	2.26
**NAP4**	AZT 10	936.87	9.49	134.17	28.55	2.97	1.88	0.31	2.04
**NAP4**	HCQ 10	1032.46	11.52	146.58	31.30	3.24	2.81	0.41	2.27
**NAP4**	LOP 5	1027.91	9.60	138.93	32.69	3.02	2.60	0.46	1.95
**NAP4**	RIT 5	868.21	7.84	91.88	22.92	2.26	2.01	0.41	1.40
**NAP5**	Control	1.38	0.29	0.45	15.87	0.62	3.77	0.17	1.31
**NAP5**	CLA 10	1457.34	1.16	4.73	20.05	0.72	6.64	0.26	1.05
**NAP5**	AZT 10	1321.37	1.27	5.64	21.80	0.76	6.81	0.26	1.11
**NAP5**	HCQ 10	1514.97	1.22	5.49	18.48	0.90	8.08	0.31	1.17
**NAP5**	LOP 5	1363.68	1.35	4.65	16.49	0.76	6.27	0.26	1.17
**NAP5**	RIT 5	1343.49	1.10	4.24	20.12	0.72	5.53	0.24	0.97
